# Knowledge, attitudes, and practices (KAPs) on agrochemical use in small-scale farmers near Lake Tana, Ethiopia: Impacts on public and aquatic ecosystem health

**DOI:** 10.1371/journal.pone.0344438

**Published:** 2026-04-17

**Authors:** Balew Yibel Zeleke, Andualem Mekonnen Hiruy, Ermias Deribe Weldemariam, Seblework Mekonen Shegen, Tesfa Aklilu

**Affiliations:** 1 African Center of Excellence for Water Management, Addis Ababa University, Addis Ababa, Ethiopia; 2 Center of Environmental Science, Addis Ababa University, Addis Ababa, Ethiopia; 3 Kotebe University of Education (KUE), Addis Ababa, Ethiopia; 4 Institute of Water Resources, Addis Ababa University, Addis Ababa, Ethiopia; Universitas Airlangga, INDONESIA

## Abstract

Agrochemical application is essential for increasing agricultural production and achieving Sustainable Development Goal 1 to secure worldwide hunger. In Ethiopia, there is haphazard use of pesticides for agricultural intensifications. However, knowledge, attitudes, and practices (KAPs) regarding agrochemical use vary globally, especially in developing countries. This inconsistency raises significant concerns for human health and environmental degradation. Therefore this study aimed to assess the KAP of agrochemicals among communities surrounding Lake Tana. A community-based study was conducted targeting 352 small-scale farmers in selected villages, categorized by four regimes. Data were collected through face-to-face interviews and in situ observations via a pretested and semi structured questionnaire. The data were analyzed via chi-square tests and logistic regression. The findings revealed that, 100% of the respondents used pesticides on their farm, 42.3% had good knowledge, 56.5% had a positive attitude, and only 22.4% practiced well. The majority (57.1%) used banned pesticides particularly DDT, and 39% repurposed empty pesticide containers for household use. Moreover, 69% and 17% of the respondents reported that they most commonly use class II (moderately hazardous), and class III (slightly hazardous), respectively. Literate respondents were 2.329 times more likely to practice good pesticide application than illiterate respondents were (AOR = 2.329, Cl = 1.228, 4.419). The educational level and attitudes of the respondents showed significant associations with pesticide application practices according to binary logistic regression, with a P value <0.05. Poor pesticide practices were more prevalent in the Dembia and Fogera plain regimes than in the Bahirdar and Gilegel Abay regimes. Education and location (study clusters) were key factors, highlighting the need for capacity building and enforcement to protect the community and the aquatic ecosystem.

## 1. Introduction

The growing world, which is in an unprecedented state, demands agricultural intensification to end hunger, achieve food security, improve nutritional status and promote sustainable development [1]. Agricultural intensification has resulted in an increase in the production and utilization of agrochemicals such as pesticides and fertilizers, both in quantity and in kind [2]. Historically, agrochemical production, particularly pesticide production, started to increase after the 2^nd^ war [3]. It was 500,000 tons per annum in the 1950s. Recently, in the 21st century, more than 4 million tons have been produced annually worldwide [4]. According to the FAOSTAT report, worldwide pesticide consumption reached more than 4,122,334 tons per annum on average from 1990--2018. Africa represents 2.1% of the country (69,984.83 tons) [4].

In Ethiopia, pesticides were first introduced in the 1940s in the agricultural sector to protect crops from pests [5]. Its usage is increasing, and the amount of imported pesticides increased from 242 tons in 1993–4,123 tons in 2018 [4]. This is due to the increasing demand of different sectors for agricultural disciplines (crop protection, flower farming, forestry, aquaculture, etc.), the food industry (food material preservatives), processing, transportation and the health sector for the prevention of vector-borne diseases, and other industries that use imported pesticides [6]. In addition to imported pesticides, Ethiopia produces an average of 1,374.161 tons of pesticides per year, with a national capacity from the Adamitulu pesticide production industry. On the other hand, the Amhara regional state agricultural sector reported an average of 140.1 tons of pesticides supplied in the Lake Tana subbasin, which is one fourth of the country utilized.

While pesticides significantly protect crops from pests and diseases from 20 to 40% production loss [7,8], their misuse can lead to severe health and environmental crises [9,10]. The indiscriminate application of pesticides poses risks to both the public health and the health of aquatic ecosystems [11,12]. Numerous incidents have occurred due to pesticide application. For example, exposure to pesticides such as organophosphates can inhibit cholinesterase enzyme activities, resulting in cholinergic symptoms [13]. Applying pesticides at concentrations higher than the registered levels can have detrimental effects on public health and aquatic ecosystems [14]. The incidence of pesticide-induced poison is particularly high in developing countries because of poor legislative practices, gaps in farmers’ knowledge and usage, lack of personal protection during application, and skill-related issues.

Studies on the knowledge, attitudes, and practices (KAPs) regarding agrochemical use have shown that small-scale farmers, especially in developing countries, often apply pesticides inadequately. This is attributed to various factors, including illiteracy, lack of formal training [15], and negative attitudes and practices [16,17]. Some studies performed in Ethiopia have revealed that the level of knowledge, attitudes and practices of farmers require attention [18–20]. Similarly, study performed in fogera district in Ethiopia reported high knowledge gaps among experts as well as local communities [21]. However, no studies have been conducted in the surrounding area of Lake Tana, the largest lake and a significant development corridor.

In the Lake Tana subbasin watershed, agricultural practices are prevalent, leading to significant environmental challenges [22]. The rapid expansion of agricultural, coupled with inadequate management of agrochemicals, particularly from recession framings has resulted in high levels of chemical runoff. This excessive use of agrochemicals contributes to the infestation of water hyacinth [23–25] and the accumulation of pesticides in the food chain of fish species [26], potentially posing health risks to consumers. To improve public health and protect aquatic ecosystems, assessments and investigations of the level of pesticide application applied by small-scale farmers at catchment scales are needed to establish a strategic management system. In the study area, one study explained the practices of the eastern part of Lake Tana [27] but did not cover the practices and influences of the lake or major rivers surrounding it.

On the basis of the authors’ review and knowledge, there is currently no published research that captures the KAPs of farmers living near the Lake Tana shoreline concerning agrochemical use and its impacts on human health and aquatic ecosystems. Additionally, analyses of the associations between variables in KAP dynamics across sites are lacking. Therefore, this study aimed to assess farmers’ knowledge, attitudes, and practices regarding agrochemical use near the Lake Tana shoreline; identify the commonly used types of pesticides and their sources; prioritize sampling zones; and offer management recommendations.

## 2. Methods

### 2.1. Description of the study areas

**Fig 1:**
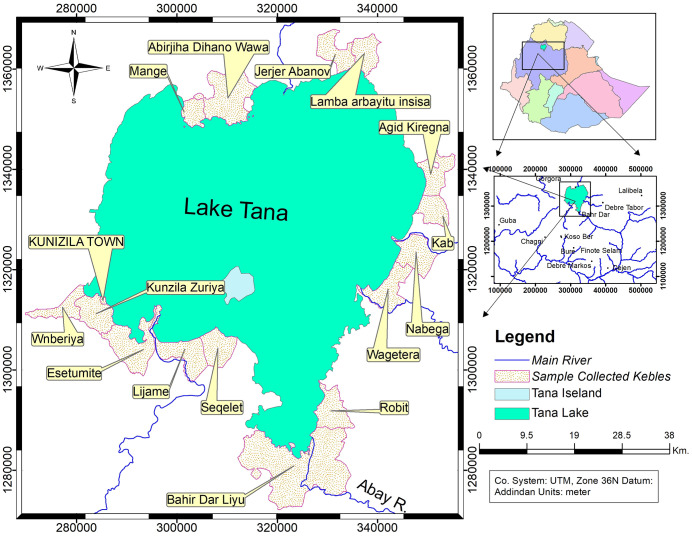
Map of Lake Tana and surrounding selected study kebeles, based on data from the Humanitarian Data Exchange [28].

The study was conducted from August to April in four selected regimes (Dembia, Fogera, Bahirdar Zuria, and Gilgel Abay) situated near Lake Tana, including the mouths of major tributaries. The study encompassed 16 kebeles, all of which are close to the lake and its five major tributary rivers (Gilgel Abay, Gumera, Rib, Megechi, and Dirma) (Fig 1).

The study areas were classified into four categories to facilitate comparison of pesticide use and agrochemical practices (Table 1).

**Table 1:** Description of the study area surrounding Lake Tana.

S.No	Study Regimes	Location	Description of the study area
1.	Dembia	North part of the Lake	The plain area is primary used for crop production from August to January.
2.	Fogera	East part of the lake	The flood plain area is predominantly used for cultivation of cereals and vegetables throughout the year, barring the major rainy season. This exception is due to the overflow of the lake and flooding, which lead to inundation of the surrounding areas.
3.	Bahirdar	South part of the lake	Mainly, residents of the town cultivate vegetables, khat and fruits year round using the lake water
4.	Gilegel Abay	South West of the lake	Mainly, rural inhabitants cultivate grains during the rainy season.

### 2.2. Sampling method and data collection procedures

A cross-sectional study was conducted using mixed-method questionnaires, which included both closed and open-ended questions to interview 352 randomly selected small-scale farmers near Lake Tana. Prior to the interviews, participants provided consent, confirming their willingness to participate either by signature or verbally. This process ensured transparency, confidentiality, and accountability throughout the research. The selected farmers primarily utilize the buffer zone of Lake Tana for recession farming, following the mouths of major tributary rivers. To identify respondents, consultations were held with regional, zonal, and district agricultural and environmental sectors to determine the necessary population size in the areas surrounding the lake and the major tributary river mouths. Four sampling regimes (Dembia, Fogera, Bahridar Zuria and Gilegel Abay) were identified, comprising 16 kebeles and specific villages that have direct contact with the river mouths and the lake. The total population of the study area was 28,000. The lists of the households that used irrigation and post rain season farming were obtained from the respective kebele managers and agricultural extension offices. The sample size was determined to be 352 on the basis of the WHO pesticide regulation protocol [29] and considering [30] following a 95% confidence interval, a 5% level of precision, and a 50% degree of variability with little modification (Equation 1).


$$\begin{array}{c} \text{n}=\text{N}/[\text{1}+\text{N}(\text{e}{)}^{\text{2}}] \end{array}$$ Equation 1

where n is the sample size, N is the total population and e is the precision.

The respondents were proportionally allocated to the four selected sampling regimes. Eight data collectors were selected and trained on the questionnaire and objectives of the study. The authors undertook the data collection process under the supervision and support of the researcher.

Local kebeles and villages, along with agricultural and health extension experts, were engaged documenting farmers’ pesticide use practices and attitudes. In addition, three key informants (KIs) from each sampling area were interviewed to provide insights into overall practices, attitudes, observed challenges, the roles of government structures and local inhabitants, and potential environmental impacts. A small focus group discussion (FGD) was undertaken to gather relevant information on pesticide handling procedures and attitudes. Observations were conducted on various aspects, including chemical mixing, leftover pesticide disposal, and their containers. Additionally, pesticide vendors were consulted to explore their role in pesticide application, the challenges they face, and the support they need from the government and regulatory bodies to contribute to the solution. These methods ensure data triangulation, rationality, and dependability. Furthermore, office data on agrochemical distributions over the past ten years, including usage frequency and related information, were compiled and integrated with the survey.

The study defined the dependent variables as the knowledge, attitudes and practices pesticide users. The independent variables included demographic information (sex, education, farming experience, land size, age, income and sampling sites), training provided, and other elements discussed during the interviews.

The questionnaire comprises five sections. The first section collected general demographic information about the respondents, including their farming experience, land tenure size, and income sources with annual revenue. The second section contained 14 questions assessing general knowledge related to crops, pesticide types, and the respondents’ understanding of pesticide use. The third section focused on aspects of agrochemical purchase, transport, and storage. The fourth section included eight questions regarding agrochemical practices. The final section featured five questions about attitudes toward pesticide management. All the questionnaires were initially prepared in English and then translated into the local Amharic language. The interviews were conducted using the translated questionnaire, which was subsequently translated to English.

The mean scores for the dependent variables were calculated. The knowledge score served as an independent variable for attitudes, whereas both the knowledge score and the attitudes score were considered independent variables for practices. The Knowledge data were recorded as ‘yes’, ‘no’, or ‘don’t know’. These responses were aggregated into a dichotomous variable, with ‘yes’ scored as one and ‘no’ and ‘don’t know’ scored as zero. A score above 50% indicated a high level of knowledge, whereas a score below 50% indicated a low level of knowledge. In addition to the independent assessment, the five-point scale data were merged into two-point scale data (positive and negative responses) for logistic regression.

### 2.3. Ethical statement

This study did not involve the collection of biological or physical samples from participants. Informed consent was obtained from all participants, either verbally or in writing, to ensuring transparency, confidentiality, and accountability throughout the research process. At the national level, there are no formal guidelines mandating ethical approval for nonclinical studies that do not involve sensitive or personally identifiable data. Since the research adhered to ethical principles including voluntary participation, confidentiality, and harms minimization, and did not process sensitive data, formal ethical clearance was not required. Nevertheless, all participants explicitly provided consent by signing the prepared consent form. For individuals who expressed their willingness verbally but were unable to provide a physical signature, their consent was documented through audio recordings. These records were securely archived as part of the ethical documentation process.

### 2.4. Data analysis

The data were organized and processed using an MS Excel spreadsheet. For the analysis, both descriptive and inferential statistics were employed with IBM SPSS version 25 software. A chi-square test was conducted to explore potential associations within the data, with significance levels set at P < 0.01 and P < 0.05. Furthermore, logistic regression was performed to assess the relationships between small-scale farmers’ knowledge, attitudes, and practices as well as demographic data and key predictor factors.

## 3. Results

### 3.1. Demographic characteristics

The demographic characteristics of the respondents are illustrated in Table 2. Over 87% of the interviewed individuals were male, which reflects the nature of their fieldwork. The majority (65.8%) were aged between 30 and 50 years, with an average age of 44 + 10 years. A significant portion of the respondents (49%) were illiterate, while 42.9% were able to read and write. Additionally, 6% had attended primary school, and 2% had completed secondary school or pursued higher education.

**Table 2:** Demographic characteristics of the respondents (n = 352).

Variables	Categories	n	Percentage (%)
Sex	Male	308	87.5
	Female	44	12.5
Age	under 30	36	10.2
	31-40	107	30.4
	41-50	126	35.8
	51-60	58	16.5
	above 60	25	7.1
Marital status	Single	16	4.5
	Married	328	93.2
	Widowed	7	2.0
	Divorced	1	0.3
Educational level	Unable to read and write	173	49.1
	able to read and write	151	42.9
	Primary school	21	6.0
	Secondary school	4	1.1
	College	3	0.9
Farming experience	1-3 Year	5	1.4
	4-10 Year	29	8.2
	11-20 Year	126	35.8
	above 20 Year	192	54.5
Income in dollar per annum	1-250	52	14.8
	251-500	130	36.9
	501-750	75	21.3
	751-1000	40	11.4
	Above 1000	55	15.6
Family size	1-2	40	11.4
	3-5	122	34.7
	above 5	190	54.0
Land holding size (ha)	Below 0.5	38	10.8
	0.5-1.00	116	33.0
	1.00-2.00	162	46.0
	Above 2.1	36	10.2

Furthermore, 54.5% of the farmer respondents had been engaged in farming activities for more than 20 years. More than 58% of the villagers reported an annual income between 250and 1000 dollars, with average revenue of 837 dollars. Nearly half of the respondents (46%) had land holdings ranging from 1 to 2 hectares, with an average of 1.44 hectares.

### 3.2. Identified type of pesticides in the study area

Twenty-eight pesticide active ingredients were identified from the small-scale farming practices interviews during the survey period (Table 3). Among them, 3.4% of the pesticides were class I (highly hazardous), 69% were class II (moderately hazardous), 17% were class III (slightly hazardous), and the remaining 10% were class V (unlikely to cause hazards in normal use) according to the WHO pesticide toxicity classification [31]. Importantly, no farmers responded to the use of pesticides of any class la.

**Table 3:** Dominantly used pesticides in the study area.

PESTICIDES TRADE NAME	ACTIVE INGREDIENTS AND COMPOSITION SUPPLIED	CLASS OF TOXICITY
AGROLAMBACIN	Profenophos 30% + Lambda Cyhalothrin 1.5%	II
AGROLAXYL	Matalaxyl 75 G/kg And Mancozeb 560 g/kg	III & U
AGROSET	Glyphosate Isopropyl Ammonium 48%	III
AJANTA	Profenofos 40% + Cypermethrin 4% E.C.	II
APRON STAR 42 WS	Thiamethoxam 20% + Metalaxyl-M 20% + Difenoconazole 2%	III
BRAVO® 500	Chlorothalonil	U
CANZOLE	Propiconazole 25% EC	II
CONFIDENCE	Imidacloprid 35% WV	II
DDT	P,P’-DDT	II
DIAZINON 60% EC	O, O-diethyl- O-(2-isopropyl-6-methyl-4-pyrimidinyl) phosphorothioate.	II
DIMETHOATE	Dimethoate 40% Ec	II
2,4 D AMIN	2,4-Dichlorophenoxyacetic Acid 41% Fl	II
ENDOSULFAN 35 EC	Endosulfan	II
GLAYCEL 41% FL	Glyphosate 41 FL	III
GLYPHOSATE 48% FL	Glyphosate 48% Fl	III
KARATE 5% EC	Lambda-Cyhalothrin	II
MALATHION 50% EC	Malathion	III
MANCOZEB 80% WG	Mancozeb	U
MATICO	Matalaxyl 80 G/kg And Maconzen 640 g/kg	III
MEGABIN 20 EC/TRICEL	Chlorpyrifos 20% Ec	II
CHLOROCEL 48 EC	Chlorpyrifos 48% Ec	II
POWER 2,4 D	2, 4 D Amine Salt 86%	II
PARAQUAT DICHLORIDE	Paraquat	II
PREMAGRAM GOLD	290 G/L S-Metolachlor + 370 G/L Atrazine	II & U
PROFIT	Profenofos 72%	II
PROFIX	Profenofos 40% + Cypermethrin 4% E.C	II
PROMAX 20% EC	Propoxur	II
RIDOMIL MZ 68% WG	Metalaxyl-M	III
ROUNDUP	Glyphosate	U
SRIZOLE/TRIAZOLE	Propiconazole 25% EC	II
SUPERPHOSE	Aluminum Phosphide 56%	Not classified
TILT 250 EC	Propiconazole	II
UNIVAN	Vancomycin	Not classified
WEED CANCEL	2, 4 D Amine Salt 72%	II
ZINC PHOSPHIDE	Zinc Phosphide	Ib

**Fig 2:**
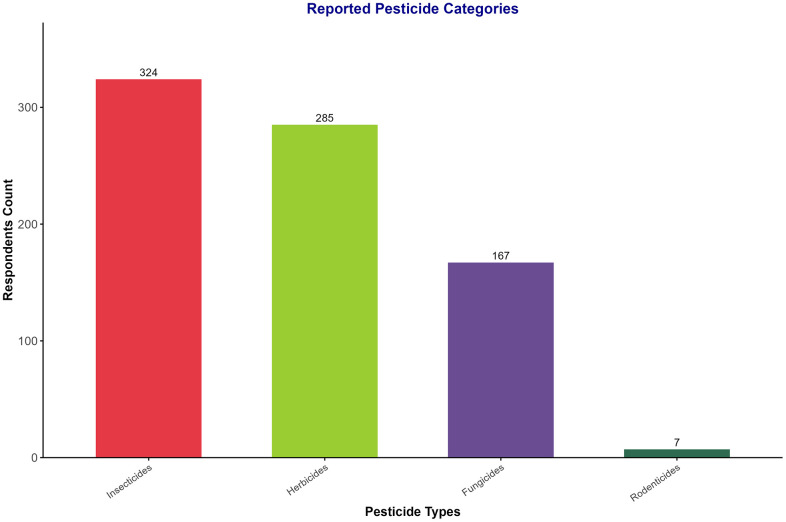
Self-reported categories of pesticides used by farmers in Lake Tana surrounding.

Fig 2 illustrates the types of pesticides used by small-scale farmers in the study area. The majority of the interviewed farmers (92%) used insecticides, whereas 81% used herbicides. Additionally, nearly half of the farmers (47.4%) utilized fungicides, and a smaller percentage (19.9%) used rodenticides.

### 3.3. Level of knowledge and understanding of pesticide application

The level of knowledge and understanding among farmers regarding the identity, danger, effects, and routes of exposure to pesticides were analyzed (Table 4). According to the survey, 95.5% of the farmers reported that pesticides have impacts on human health, and 84.4% believed that they have impacts on the aquatic environment. Among the total respondents, 42.7% did not have any information about banned pesticides. Additionally, 64.5% of the farmers were unable to identify the color code of pesticides, and 73.6% did not read, understand, or follow the labels on the containers. The interviewed farmers considered spraying (80%), mixing (29%), storing (20%), and reentering treated areas (11%) as potential routes of pesticide exposure. Similarly, they reported dermal (71.6%), oral (78.6%), inhalation (52.6%), wound (44.9%), and ocular (42.9%) routes of pesticide exposure (Table 4).

**Table 4:** Farmers’ knowledge of pesticide application.

Variables		Frequency	%
**Do you think that pesticides affect human health?**	Yes	336	95.5%
	No	16	4.5%
**Do you think that pesticides can have a negative impact on the aquatic environment?**	Yes	297	84.4%
	No	22	6.3%
	Don’t know	33	9.4%
**Do you know the banned lists of pesticide?**	Yes	201	57.1%
	No	151	42.9%
**What are the reasons of banning or restricting those aforementioned pesticides** ^**a**^**? (n = 201)**	High toxicity	72	35.6%
	Expensive	4	2.3%
	Not effective	123	60.9%
	Don’t know	3	1.5%
**Are you aware of the color codes of pesticides and sign of toxicity on the containers?**	Yes	125	35.5%
	No	227	64.5%
**Do you read, understand and follow the labels of the containers?**	Yes	93	26.4%
	No	259	73.6%
**Did you take training on pesticide application?**	Yes	116	33.0%
	No	236	67.0%
**How do you see the level of danger of pesticides to work with?**	Very dangers	217	61.6%
	Same what dangers	96	27.3%
	Less dangers	33	9.4%
	Not dangers	6	1.7%
**Which activity could be the cause of possibilities of exposure to pesticides?** ^**a**^	Mixing	103	29%
	Spraying	283	80%
	Transportation	36	10%
	Setting	70	20%
	Taking care of equipment	30	8.50%
	Reentering in to sprayed	39	11%
	Disposal	30	8.50%
**In which major routs/ways, pesticides enter into human body?** ^**a**^	Through skin(Dermal)	252	71.60%
	Through nose(Inhalation)	185	52.60%
	Through mouth (Oral)	171	78.60%
	Eye (Ocular)	151	42.90%
	Wound	158	44.90%

^a^more than one response was reported (not mutually exclusive)

**Fig 3:**
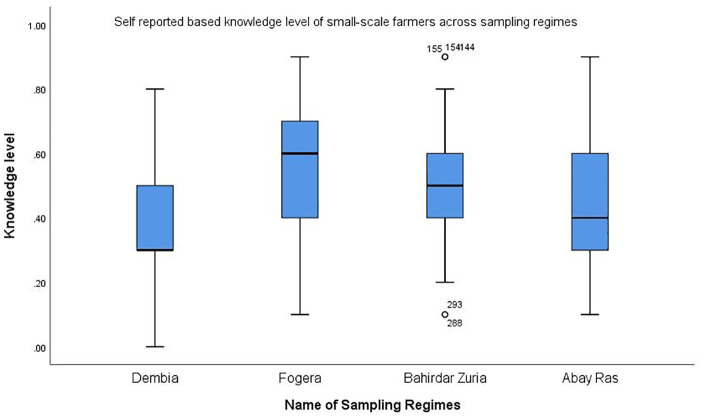
Observed level of knowledge at Lake Tana surrounding farmers.

Fig 3 shows the knowledge scores of the four study regimens. Fogera and Bahirdar Zuria achieved scores of 0.57 and 0.51, respectively, which were above the average of 0.5. In contrast, Dembia and Gilgel Abay obtained scores of 0.43 and 0.37, respectively, which were below the average, which implies that the Fogera and Bahirdar Zuria study regimens had relatively better knowledge than the Dembia and Gilgel Abays did.

### 3.4. Levels of pesticide application and use practices

#### 3.4.1. Sources of pesticides and application practices.

**Fig 4:**
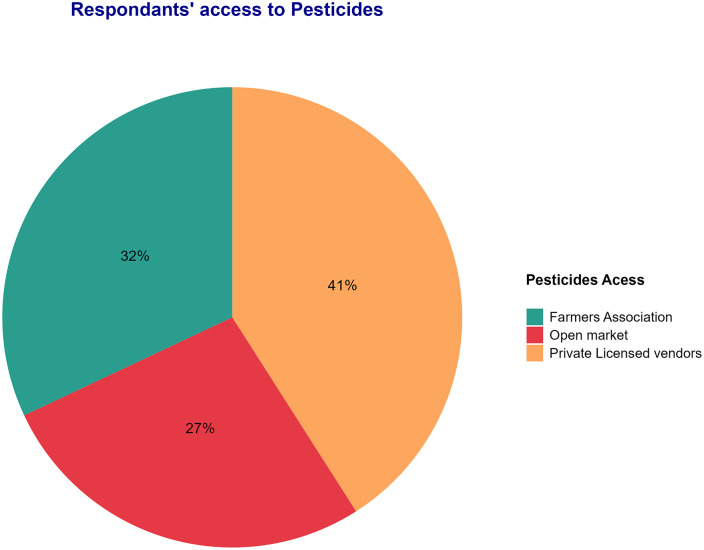
Retail pesticide access points observed in local market settings.

All the respondents used pesticides on their farms to eliminate pests (Table 5). Most farmers (72%) consulted agricultural experts when choosing a pesticide, whereas 57.6% received advice from pesticide vendors. However, nearly 20% of the interviewed farmers applied pesticides without seeking any information. In terms of pesticide sources, 41% of the respondents obtained them from private vendors, 32% from farmer associations, and 27% from the open market. These findings are based on a sample that represents 100% of the suppliers, as shown in Fig 4.

**Table 5:** Pesticide application practices of small-scale farmers (n = 352).

Variables Practices of the Small scale farmers		Frequency	%
**Do you use pesticides in your farm to protect the crops from pests?**	Yes	352	100%
	No	0	0%
**Whom do you recommend to choose a particular pesticide?** ^**a**^	DAs (Development Agents)	254	72.0%
	Pesticide suppliers/vendor	203	57.6%
	Other farmers experiences	81	23.0%
	Your own Previous experience	75	21.0%
**How frequently are you handling and using pesticides?**	Throughout the year	63	17.9%
	Tow season per year	175	49.7%
	One season per year	107	30.4%
	Others	7	2.0%
**Which way do you prefer to take care (safe) during spray?** ^**a**^	Don’t spray against wind	28	8.0%
	Don’t spray during raining	49	13.9%
	Spray at morning and afternoon	264	75.0%
	Spray any time	15	4.3%
**What is your source of pesticide handling instructions?**	Labels on the container	63	17.90%
	Pesticide Vendor	158	44.90%
	Agricultural extension workers	123	35%
	Without consulting	15	4.30%
**What is your experience looking at the labels & instructions on pesticide containers?**	Read & but not understand	102	29.0%
	Read, understand & follow	93	26.4%
	could not read & understand	147	41.8%
	Bought pesticides without labels	10	2.8%

^a^more than one response was reported (not mutually exclusive).

Most small-scale farmers use knapsack sprayers for mixing and spraying pesticides. These knapsacks were readily available on the market. Approximately 78.4% of the respondents applied pesticides three to ten times per cropping season, depending on the crop type and location. Moreover, 17% of the plants were treated with pesticides one to two times per cropping season, and 4.5% were treated with pesticides 10–25 times per cropping season.

With respect to the timing of pesticide spraying, the majority of farmers (75%) sprayed pesticides in the morning and afternoon, taking into consideration factors such as wind (8%) and rain (13.9%). However, 4.5% of the interviewed farmers sprayed pesticides at any time without considering the means of protecting their health or the environment.

A small proportion of the interviewed farmers (17.9%) followed the instructions and labels on the pesticide containers. There was a significant association between following pesticide handling instructions and farmers’ knowledge scores (P < 0.05).

#### 3.4.2. Pesticide transport, storage, leftover and empty pesticide container management and disposal practices.

Pesticide transportation, storage, handling, and disposal practices are illustrated in Table 6. The majority of the farmers interviewed (88.1%) transported pesticides by human beings. A significant portion (33.8%) carried them along with household goods, and among these, 30% did not take any precautions.

**Table 6:** Pesticide transport, storage and disposal practices of small-scale farmers.

Variables		Frequency	%
**What kind of methods you use to transport pesticides from the market?**	Cart	11	3.1%
	open truck	2	0.6%
	bus	29	8.2%
	Animal (donkey) back	0	0.0%
	Human by carrying	310	88.1%
**Do you regularly or occasionally transport pesticides with other goods such as food items?**	Yes	119	33.8%
	No	233	66.2%
**If yes, what precautions do you take to prevent and control pesticide contamination? (n = 119)**	None	30	25.0%
	Cover checking	68	57.0%
	Separated setting	21	18.0%
**Where do you store pesticides before use it?**	Separated place	104	29.5%
	Anywhere in the house	234	66.5%
	Animal living house	5	1.4%
	Use as soon as purchasing	16	4.5%
**Where do you store pesticides’ spraying equipment?**	General storage within the house	20	5.7%
	Equipment store	48	13.6%
	Ceiling board	202	57.4%
	Bedroom	35	9.9%
	Others	47	13.4%
**How do you dispose the unwanted/leftover or no longer in use pesticides?**	Dispose by incineration/Burning	27	7.7%
	Spill over the nearby water bodies and farm	155	44.1%
	Selling to other body	13	3.7%
	Use for other crops	21	6.0%
	Dispose by Landfill	136	38.6%
**How do you dispose empty pesticide container after uses?**	Use for House purpose	137	39%
	Disposing on the farm & to nearby water body	129	36.70%
	Separated area	90	25.50%
	Selling to Koralio	60	17%

Only 29.5% of the respondents stored pesticides in a separate location inaccessible to family members. In contrast, 66.5% stored them anywhere in the house, with 1.4% keeping them in the animal house, and 4.5% using them immediately after purchase (Table 6). Additionally, 57.4% of the respondents stored pesticide spray equipment on a ceiling board without ventilation, and 10% kept it in the bedroom.

**Fig 5:**
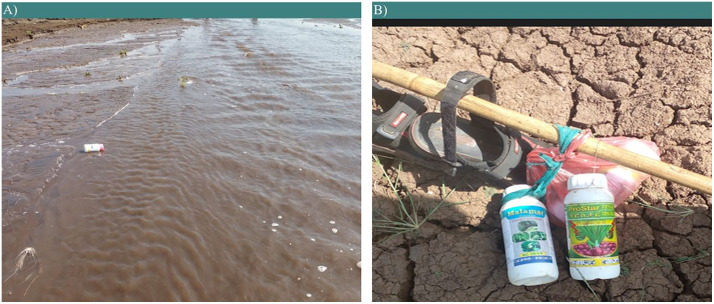
Pesticide container management practices in Lake Tana surrounding farmers: (A) Improper empty pesticide containers disposal practices near the mouth of the Megech River; (B) Empty pesticide containers used for reuse purposes, Photo by the author.

Nearly half of the interviewed farmers (44.1%) disposed of leftover pesticides by pouring them into nearby water bodies. A considerable respondent (38.6%) disposed of pesticides that potentially contaminate ground water. Additionally, 6.0% of the respondents used the leftover pesticides on other crops, while 3.7% sold them to other farmers. Regarding empty containers, a significant portion of respondents (36.7%) disposed of them on farms or in nearby water bodies (Fig 5A). Additionally, 39% of the respondents reported repurposing them for home (Fig 5B), while 25.5% disposed of them in designated areas, including burning sites. The remaining 17% sold the containers to Kororalio.

#### 3.4.3. Personal protective equipment (PPE) use practices.

**Fig 6:**
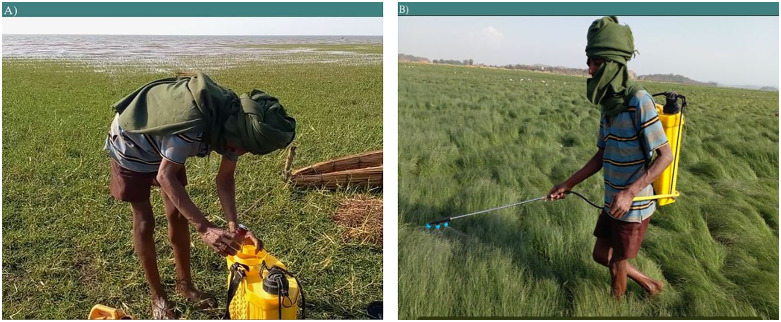
Pesticide mixing and spraying practice in Lake Tana surrounding farmers: (A) Pesticide mixing practices observed during fieldwork, demonstrating local preparation methods, (B) Spraying practices conducted in the recession agricultural zones within the Fogera regime.

In the present study, none of the small-scale farmers used standard personal protective equipment while preparing, mixing, spraying, or disposing of pesticides and their containers as illustrated in (Fig 6A and B). However, some farmers reported taking minimal precautions: 7.4% covered parts of their body with local clothing, 28.1% partially covered their faces, and 29.3% sporadically wore protective items such as boots, trousers, and gloves. Notably, 35.2% of the interviewed farmers reported not using any protective devices at all. This lack of protective measures was found to be significantly associated with knowledge and education levels (P value <0.001).

### 3.5. Reported public health symptoms and environmental impacts regarding pesticide application

**Fig 7:**
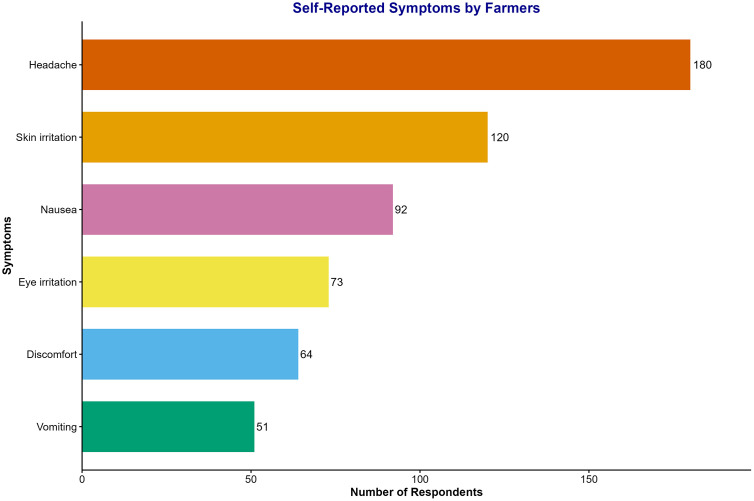
Self-reported symptoms by farmers after pesticide application.

Most of the farmers interviewed believed that pesticides are harmful to human health (95.5%) and aquatic ecosystems (84.4%). Small-scale farmers reported varying levels of pesticide exposure and experienced different symptoms, including headaches (51.4%), skin irritation (41.8%), nausea (31.3%), eye irritation (24%), discomfort (24.1%), and vomiting (10.2%) during pesticide application (Fig 7). The effects of these symptoms lasted for more than 24 hours. Only 24.7% of the respondents sought treatment at a nearby health post; 24.4% opted for self-treatment and 9.4% did not seek any treatment. Additionally, 6.8% used traditional medicine, and the remaining cases may not have been serious enough to warrant treatment.

**Fig 8:**
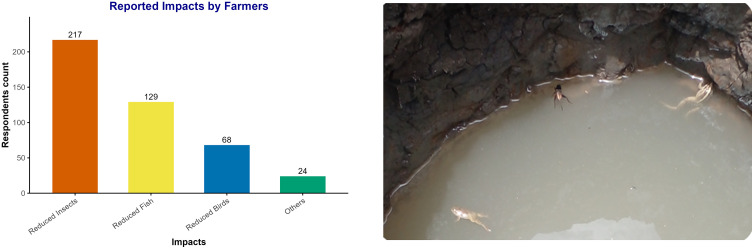
Reported Pesticide impacts during the study (A) Self-reported impacts of pesticide application on biodiversity reduction; (B) Observed local impacts of pesticides use on agricultural land near lake wells, visible death of biota.

All farmers in the study area relied on lake water and major tributary rivers flowing into the lake to prepare pesticide mixtures and clean their equipment. Additionally, approximately half of farmers (44.1%) reported disposing of leftover pesticides and empty containers in nearby water bodies. Many farmers in the surveyed community noted the impacts of pesticides on aquatic ecosystems. Specifically, 61.6% of the farmers reported a reduction in beneficial insects, particularly honey-making bees; 36.6% reported a decline in fish populations and other aquatic organisms; and 19.3% noted impacts on birds (Fig 8A). These ecological changes are attributed primarily to the extensive use of pesticides on farms and their effects on surrounding water bodies. The authors also documented the harmful effects of pesticide application on farm ponds and wells, which resulted in the death of aquatic organisms (Fig 8B). Moreover, 16.2% of the respondents reported an increase in the contamination of fish and other aquatic organisms, whereas 5.6% reported pollution of the lake water. Notably, 10% of the farmers did not believe that pesticides have any impact on the environment.

### 3.6. Associated factors of farmers’ attitudes toward pesticide application

The attitudes of small-scale farmers toward the safe application of pesticides were significantly associated with several independent variables: sex, educational level, sampling regime, knowledge level, awareness of the negative impacts of pesticides on aquatic ecosystems and human health, and pesticide safety on the market (Table 7). This association was determined using a binary logistic regression model, with a P value of less than 0.05.

**Table 7:** Factors associated with the attitudes of small-scale farmers toward safe pesticide application.

Factors associated		Attitudes of farmers		Crude Odd Ratio		Adjusted Odd Ratio	
		Negative	Positive				
		N	N	OR	95% Cl	OR	95% Cl
Sex	Male	121	187	0.243	0.12, 0.49	0.417	0.185, 0.94*
	Female	32	12				
Educational level	Illiterate	106	67	4.443	2.83, 6.98	3.065	1.85, 5.08*
	Literate	47	132				
Sampling Regimes	Dembia	52	36	0.916	0.76, 1.11	0.966	0.775, 1.21
	Fogera	22	66				
	Bahirdar Zuria	20	68				
	Gilegel Abay	59	29				
Knowledge level	Low	115	69	5.702	3.57, 9.11	2.623	1.469,4.69*
	High	38	130				
Aware of the negative impacts of pesticides on Aquatic Ecosystem	Yes	59	148	4.623	2.93, 7.29	2.403	1.352, 4.27*
	No	94	51				
Pesticide effect on human health	Yes	146	190	0.988	0.36, 2.72	0.458	0.135, 1.56
	No	7	9				
Pesticide in the market are safe	Yes	42	83	0.529	0.34,0.83	0.601	0.354, 1.02*
	No	111	116				

*Statistically significant at P value < 0.05.

The model demonstrated that individuals within the literate community, ranging from those who can read and write to those with a college education, were 3.065 times more likely to hold positive attitudes than negative attitudes (AOR: 3.065, 95% CI: 1.85--5.076). Farmers who were knowledgeable about pesticide application were 2.623 times more likely to have positive attitudes toward pesticide use than those with limited knowledge (AOR: 2.623, 95% CI: 1.469–4.685). Additionally, respondents aware of the negative impacts of pesticides on aquatic ecosystems were 2.103 times more likely to maintain positive attitudes toward pesticide use than those who were unaware of these impacts, with a 95% confidence interval (CI) of 1.352--4.268, indicating that the true odds ratio is likely around 2.403 (AOR = 2.403, 95% CI; 1.352--4.268).

### 3.7. Factors associated with farmer’s pesticide application practices

The study revealed significant associations between respondents’ sex, sampling regime, educational level, and attitudes toward pesticide application practices, as indicated by binary logistic regression (P < 0.05). In contrast, factors such as small-scale farmer knowledge, exposure during mixing and disposal, farming experience, and annual income were not significantly linked to pesticide application practices in the study area (Table 8).

**Table 8:** Factors associated with small-scale pesticide application practices among the interviewed farmers.

Variables		Level of Practices		COR (95% CI)	P Value	AOR(95% CI)	P Value
		Poor	Good				
		No	No				
Sex of respondent	Male	232	76	0.223(0.067, 0.742)	0.014	0.253(0.071, 0.903)	0.034*
	Female	41	3				
Name of Sampling Regimes	Dembia	76	12		0.00		0.002
	Fogera	75	13	1.098 (0.471, 2.561)	0.829	0.474(0.168, 1.338)	0.158
	Bahirdar Zuria	62	26	2.656 (1.24, 5.689)	0.012	1.353(0.519, 3.528)	0.536
	Gilgel Abay	60	28	2.956 (1.387, 6.296)	0.005	3.913(1.540, 9.945)	0.004*
Educational Level	Illiterate	151	22	3.206 (1.856, 5.54)	0.000	2.329(1.228, 4.419)	0.008*
	Literate	122	57				
Attitude of the small scale farmers	Negative attitude	134	19	3.044(1.725, 5.372)	0.000	2.387(1.155, 4.932)	0.019*
	Positive attitude	139	60				
Exposure from mixing to disposing	No	205	56	1.238(0.709, 2.162)	0.453	2.155(0.967, 4.801)	0.061
	Yes	68	23				
Knowledge general level	Low knowledge	154	30	2.114(1.265,3.532)	0.004	1.395(0.695, 2.802)	0.349
	High knowledge	119	49				
Farming Experience	Mean	25	26	1.012(0.99, 1.034)	0.291	1.013(0.988, 1.039)	0.300
Income of Small scale Farmers in dollar	1-250	37	15		0.007		0.236
	251-500	112	18	0.396(0.182, 0.864)	0.020	0.467(0.172, 1.264)	0.109
	501- 750	61	14	0.566(0.246, 1.305)	0.182	0.565(0.183, 1.741)	0.320
	751-1000	28	12	1.057(0.28, 2.611)	0.904	0.730(0.22, 2.421)	0.607
	Above1000	35	20	1.409(0.625, 3.18)	0.408	1.138 (0.366, 3539)	0.823

* Statistically significant at P value < 0.05.

Literate respondents were 2.329 times more likely to engage in proper pesticide application than illiterate respondents were (AOR = 2.329, CI = 1.228, 4.419). Those with positive attitudes toward pesticide use were 2.387 times more likely to practice good methods than those with negative attitudes were (AOR = 2.387, CI = 1.155, 4.932). Gender also had a significant effect, with an adjusted odds ratio of 0.262 (CI = 0.074, 0.933). With respect to the sampling regimes, respondents from Fogera were 0.474 times less likely, those from Bahir Dar were 1.353 times more likely, and respondents from Gilgel Abay were 3.913 times more likely to demonstrate good practices than those from Dembia.

A logistic regression model was employed to evaluate the influence of demographic factors (sex, education level, annual income, and sampling regimes) and additional factors, such as knowledge level, attitudes, and basic practices, on farmers’ adoption of good pesticide application practices. The analysis included 352 respondents and explained 25.8% of the variation in pesticide application practices, correctly classifying 83.5% of the cases.

## 4. Discussion and implications for pesticide use practices

Assessing farmers’ level of knowledge, attitudes, and practices related to agrochemical use is vital for revitalizing policies, strategies, legal frameworks, and management systems that aim to minimize and mitigate the health impacts on humans and aquatic ecosystems caused by the misuse of these chemicals [32]. In the present study, 95% of the farmers acknowledged the negative impacts of pesticides on human health, whereas 84% recognized their negative impacts on environmental health. Similar findings have been reported in other countries, with 92% in Uganda [33], 90% in Nepal [34], and 71% in Kuwait [12]. Despite this awareness, only 26.4% of farmers in the study area understood and followed safe pesticide-handling practices. This finding aligns with a study by Mekonnen and Agonafir [15], which revealed that 33% of respondents in Rift Valley lakes read labels and followed safety instructions. In contrast, research by Damalas and Koutroubas [35] reported that 52% of farmers adhered to label guidelines and safety practices.

Nearly half of the farmers (49%) living around the lake are illiterate, which limits their ability to understand labels and instructions on pesticide containers [36,37]. Furthermore, 67% of these farmers had not received any training in pesticide application. This lack of training may be attributed to insufficient trained personnel at lower levels of the Ethiopian government, as reported by Mergia *et al.* [18]. The key informants interviewed at each kebele also noted that both local and regional governments have not adequately addressed pesticide use and application. They emphasized that initiatives and programs to support small-scale farmers, particularly around the lake, have not been introduced in the subbasin. On the other hand, the focused group discussion confirmed that capacity-building initiatives and programs have not included farmers living around the lake that use pesticides. Participants explained that responsible bodies have not reached out to them, citing the government focus, the distance from the lake surrounding, and accessibility issues related to district and local offices. Meanwhile, the Crop Development and Protection director from the regional agricultural bureau stated that a capacity-building program is currently being trialed across the region through training of trainers (ToT) initiatives at the regional level, which then cascades down to local structures, and training at the community level. He also noted that, beyond recommending market access for input supplies, little work has been done in the lake subbasin. Despite these efforts, the program has not fully addressed the needs of all stakeholders and may not reach the areas surrounding the lake due to limited resources and the diverse needs of agrochemical users.

Farmers with formal education tend to pay closer attention to supplier recommendations, labels, instructions, and safety measures [17]. However, suppliers often lack the necessary expertise to effectively advise farmers on pesticide applications and usage. In fact, the majority of the farmers in the study area (60.2%) relied on pesticide suppliers for guidance on the type, dosage, and frequency of pesticide application. Research conducted in various countries, including Nepal [34], Kuwait [12], Ethiopia [38], and India [39], have revealed that pesticide vendors are the primary source of information regarding pesticide use for farmers. Unfortunately, the knowledge and attitudes of pesticide suppliers are often overlooked, leading to misunderstandings and misuse of pesticides, which poses risks to both human health and the environment. A study in developing countries by Desye *et al.* [40] found that 56.9% of pesticide venders could not adequately explain the required dosage, safety instructions, or precautions to farmers. Therefore enhancing their knowledge and attitudes and enforcing regulations that require pesticide suppliers to meet established standards are important. This will ensure that they provide farmers with comprehensive information on pesticide application, including transportation, storage, and usage.

A total of 88.1% of the farmers reported that they transport pesticides themselves. Of these, 25% admitted to carrying pesticides alongside food items without taking any precautions, which poses a risk of food contamination and potential harm to children. The act of transporting pesticides alongside food and other heavy objects or materials can cause spillage and contaminate the surrounding area, including humans [41]. Furthermore, the study revealed that 66.5% of the respondents stored pesticides anywhere in their houses, which became a source of danger. Poor storage practices led to 4.5% of the respondents experiencing death as a result, although these incidents were not intentional. Similar findings were reported in a study conducted in Tanzania [17]. These inadequate storage practices contribute to numerous incidents of injury, death, and health risks for both humans and animals. In many developing countries, farmers’ storage practices are generally poor and pose a significant potential source of exposure, particularly for vulnerable family groups [17].

Farmers consistently used pesticides classified as WHO classes II and III, such as chlorpyrifos, diazinon, lambda-cyhalothrin, profenofos, propiconazole, and glyphosate, across all four study regimes. Similarly, research conducted in Uganda [33] and Ethiopia near Zeway Lake [18] revealed that farmers rely mainly on class II and III pesticides. In contrast, this study revealed that 3.4% of the farmers reported using one pesticide from class Ib (hazardous) or zinc phosphide. In comparison, 69% reported the use of two banned or restricted pesticides, DDT and endosulfan, which are classified as class II (moderately hazardous). Importantly, the US EPA classifies DDT and endosulfan, as class I because of their high acute toxicity from oral or dermal exposure. The use of banned DDT and endosulfan is common practice in developing countries. A case study conducted by Plianbangchang *et al.* [42] revealed that farmers mainly use banned endosulfan pesticides on their farms. This may be due to a lack of community awareness. The farmers indicated that they use the banned pesticides DDT and endosulfan because they are more effective at eliminating pests than other available options. While 57.1% of the respondents were aware of the existence of banned pesticides, only 32.7% understood that these restrictions were due to their toxicity. Most farmers purchase banned pesticides without fear of the rules, even though the vendors supply them in a concealed way. The farmers justified the use of banned pesticides by citing their high effectiveness in removing pests. Farmers’ perceptions lead them to accept the risk associated with banned pesticides without fully understanding the risks they pose to humans and the environment. A study conducted by Negatu *et al.* [19] reported that 94% and 25% of small-scale irrigation farmers in Rift Valley, Ethiopia, used endosulfan and DDT, respectively. In contrast, according to Mergia *et al.* [18], small-scale irrigation farmers in the same study area stopped using DDT and endosulfan. This change is attributed to the effectiveness of the National Implementation Plan at the local level disseminated by [43].

The frequency of pesticide spraying varies by crop and regime. In the study area, 62.5% of the farmers reported applying chemical pesticides at least five times per crop per season, whereas 17.9% reported doing so less frequently. Nearly half of the respondents (49.7%) indicated that they sprayed twice a year. The factors influencing the frequency of spraying include crop type, pest prevalence, agro-climate, environmental conditions, and pest management practices. Notably, 2.3% of the farmers reported spraying more than 20 times, particularly for khat. This practice significantly increases pesticide application in subbasins, posing risks to both human health and aquatic environments.

In the study regimes, the majority of the farmers were aware of the routes of agrochemical exposure, including skin contact (71.5%), inhalation (52.5%), and ingestion (48.5%), which aligns with the findings of other studies conducted in Tanzania and Ghana [17,44]. The most common health-related symptoms resulting from agrochemical exposure, including headache, nausea, vomiting, skin irritation, eye irritation, discomfort, and even death in special cases, are reported by small-scale farmers. These events and symptoms could be the result of contact with active pesticide ingredients, including the inhibitory factor acetylcholinesterase [45].

During the authors’ field observations, it was noted that most farmers were preparing pesticides near major rivers, close to rivers, at the confluence of rivers and lake water and lake itself. These practices were further validated through a focused group discussion, which confirmed that farmers use the nearby river and lake water for preparations of sprayed pesticides, washing and disposing the left over pesticides due to lack of understanding the consequence and regulations. These practices can harm aquatic ecosystems, threaten endemic fish species in lakes and pose risks to human health. Some studies conducted in Lake Tana and associated wetlands and tributary rives have detected hazardous pesticides in the water and fish. For example, Abera *et al.* [26] reported the presence of DDT, DDE, Cypermethrin, and Chlorothalonil in Lake Tana and its wetlands. Another study focused on fish, sediment, and water in Lake Tana identified hazardous pesticides such as endosulfan, DDT, DDD, DDE, deltamethrin, and lindane [46], which are also mentioned in this study interviews with local farmers. Furthermore, a study by Zeleke *et al.* [47] indicated that the release of agrochemicals, including pesticides, into the Lake Tana water is linked to elevated levels of potentially toxic elements (PTEs) associated with pesticide use. High levels of pesticide residues in ecosystems can disrupt the food chain, and community practices are likely direct contributors to the presence of these pesticides in the lake. While, only nearly one-fourth of the community recognized a decline in aquatic biodiversity, including fish and beneficial insects, in the lake and surrounding rivers. In contrast, a study conducted around Lake Zeway, Ethiopia, reported that 80% of surveyed farmers acknowledged a decline in biodiversity [18]. In the study area, farmers (73.6%) predominantly prepared pesticide mixtures on the basis of personal experience, guesswork, or information from vendors, often disregarding official instructions. The concentrations used varied from 0.125 to 2.5 liters or kilograms per hectare and were applied five to fifteen times. This approach leads to excessive pesticide use and significant environmental contamination.

The study revealed that farmers residing near Lake Tana and the mouths of major tributary rivers presented low knowledge levels, negative attitudes, and poor pesticide application practices. Research conducted in western Bhutan [48] also indicated low levels of knowledge, significant negative attitudes, and inadequate pesticide practices, resulting in increased exposure to pesticides. These negative attitudes are closely associated with factors such as illiteracy, a lack of awareness of the full harmful effects on aquatic ecosystems, and dependence on various sampling regimes (Dembia, Fogera, Bahir Dar, and Gilegel Abay, respectively). These negative attitudes may stem from poor pesticide application methods, including neglect of personal protective equipment and misuse of pesticides, which pose risks to both humans and aquatic ecosystems. Research in Jordan reported that knowledge levels significantly influence attitudes [11], whereas a study in Kuwait corroborated that knowledge levels affect farmers’ attitudes [12]. Additionally, a very low level of pesticide application practices was strongly associated with illiteracy, negative attitudes, and varying sampling regimes. The attitudes and educational background of the farmers shaped their pesticide application practices. A study in California’s San Joaquin Valley illustrated how attitudes impact the practices of small-scale Hmong farmers [49]. Poor practices in small-scale farming can lead to immediate health issues, ranging from mild symptoms such as itchiness and skin irritation to severe effects such as nausea, vomiting, eye irritation, and respiratory problems. Moreover, these practices contribute to biodiversity loss. A systematic review of global acute unintentional pesticide poisoning cases revealed approximately 385 million incidents occur annually [50], which adversely affects both human and environmental health in this study. Thus, pesticide application practices are critical to addressing concerns regarding human and environmental health.

## 5. Conclusion and recommendations

This study provides comprehensive evidence on the knowledge, attitudes, and practices of farmers surrounding Lake Tana, Ethiopia. The study finding reveals substantially limited knowledge, predominantly attitudes, and inadequate pesticide handling and application practices across the study area. The level of knowledge and educational status were found to be key factors influencing farmers’ attitudes toward improving their safe pesticide application and practices. Farmers with higher levels of education were strongly associated with positive attitudes and safer practices, emphasizing the importance of education fostering behavioral change. Additionally, factors such as the sampling regime and the gender of the farmers were significantly related to pesticide practices. Attitudes and practices varied notably among the different sampling regimes in Dembia, Fogera, Bahirdar Zuria and Gilgel. Abay. However, farmers’ knowledge scores, farming experience, and annual income did not show significant associations with pesticides practices. The study also identified a highly toxic pesticide from Category Ib, along with two banned pesticides (DDT and endosulfan) classified as Category II. Nevertheless, the most commonly used pesticides in the study area were those classified by the WHO as categories II and III. None of the interviewed farmers used advanced PPEs, with some relying on locally sourced PPE that may not adequately protect community health. This practice poses risks of acute intoxication, long-term health effects, and potential threats to aquatic ecosystems. This study highlights the necessity for comprehensive education for farmers regarding pesticide application, appropriate pesticide levels, and the environmental impacts on public and aquatic ecosystems. Furthermore, pesticide vendors play crucial roles in providing important agricultural information to farmers. Therefore, designing and implementing capacity-building programs are indispensible for government, development partners, and academia to strengthen the systems that support pesticide suppliers, extension workers, and small-scale farmers Furthermore, agrochemical management interventions are advised to address the challenges faced in the study regimes of Dembia, Fogera, Bahirdar Zuria, and Gilegel Abays. Moreover, we strongly recommended further study to detect and quantify the agrochemical residues across environmental compartments; water, soil, air, biota as well as within human biometrics in order to understand the impact of agrochemicals on both ecosystem health and human well-being.
